# Harnessing Bourdieu's social theory to understand the deteriorating doctor-patient-nurse relationship in West Bengal government hospitals

**DOI:** 10.3389/fsoc.2022.938734

**Published:** 2022-10-06

**Authors:** Tannistha Sarkar

**Affiliations:** Department of Sociology, University of California, San Diego, San Diego, CA, United States

**Keywords:** workplace violence, government hospitals, healthcare workers, West Bengal, India, habitus, forms of capital, social space

## Abstract

Communication patterns between doctors, nurses and patients determine both the efficiency of healthcare delivery, and the job satisfaction of healthcare workers. Job satisfaction is important to ensure retention of the doctor and nurse populations. Incidents of assault against physicians and nurses from relatives and family members of patients have become frequent both in the pre-pandemic and COVID-19 eras. Along with appreciation for frontline healthcare workers serving during the pandemic, there is physical violence directed at them for failing to salvage infected patients. Using Bourdieu's concepts of social space, forms of capital, and habitus this paper endeavors to theorize some of the interaction patterns observed in doctor-patient, nurse-patient, and doctor-nurse encounters that contribute to the waning of the relationship between healthcare workers and wider society as observed in West Bengal, India. Primary empirical data was collected through in-person, in-depth semi-structured interviews with both open and closed-ended questions conducted throughout 2018 across 5 government hospitals in Kolkata (major metropolitan center) and 1 hospital in a suburban area with population 100,000. The respondents consisted of 51 nurses (100% women), 20 doctors (5% women), and 33 patients (33.3% women) recruited using purposive and snowball sampling. Social space analysis indicated that the cumulative patient social capital is comparable to that of the doctors, despite the doctor's higher levels of cultural and economic capital because of the high patient to doctor ratio. The patient population can thus concentrate and delegate their social capital to select agents leading to violence against healthcare workers. Through this analysis, two doctors' habitus were postulated, along with a nurse and a patient habitus. The first doctor habitus is structured by the idealized status of doctors and the second habitus is structured by their resource-limited working conditions. The nurse habitus is structured by the desire for economic empowerment along with dutifully providing care as instructed. The patient habitus is structured by the need to balance healthcare expenditures with their limited financial means. This paper establishes how the habitus of the agents and the politics of healthcare interact to exacerbate extant tensions between healthcare workers and the population they care for.

## Introduction

“*It will be a long discussion beyond the scope of this questionnaire. As the doctor deals with life and death of a human being, he was falsely projected as a demi-god in the past. With the advent of information boom through the internet, and catalyzed by yellow journalism, common man nowadays ‘knows-all'. The myth was shattered. The mutual respect gone. Distrust deepens. The market economy also plays an important role in widening the gap. The eternal blame game continues. The doctors are put up in the docks whenever he fails. This is a vicious cycle. The society suffers”*—Dr. M Thakur (name changed).

Amidst the government-initiated doctor worship to support doctors during the COVID-19 pandemic in India (Roy, 2020), there continues to be cases of physical violence directed at healthcare workers and doctors in India, and in West Bengal (Basu, 2019a). Incidences of violence against healthcare workers is not specific to the pandemic but has been a longstanding occupational hazard (Gates et al., 2011; Anand et al., 2016; Brophy et al., 2018; Basu, 2019a,b; Dhillon, 2019; Lindquist et al., 2019; Telegraph Bureau, 2019; Davey et al., 2020; Kesavan et al., 2020). While the issue of healthcare workplace violence in West Bengal remained under the radar until a doctor's strike in 2019, it has been a part of the healthcare system for a long time.

In June 2019, in reaction to the death of a 75-year-old patient in Kolkata's Nil Ratan Sircar (NRS) Hospital, a mob of about 200 men allegedly physically assaulted the junior doctors working the night shift in the hospital. One of the doctors suffered traumatic brain injury as a result of this attack (Telegraph Bureau, 2019). This was followed by strikes by doctors at state and national levels to protest this violence (Dhillon, 2019). The hashtags #NRSHospital, #DoctorsProtest and #DoctorsFightBack started trending in local news media. Alleged perpetrators of this attack were arrested and booked under 5 separate charges under the Indian Penal Code. They were however released for lack of evidence with a meager ₹2,000 (US $27) bail each (Express News Service, 2019).

Physical violence directed at healthcare workers is not isolated to West Bengal. Doctors working in another Indian state of Tamil Nadu also report physical workplace violence (Kesavan et al., 2020). Similarly, North American healthcare workers in the province of Ontario, Canada report violence, and highlight the lack of enthusiasm of hospital management in combating the issue as a contributing factor to the violence (Brophy et al., 2018). Finally, the job dissatisfaction of doctors caused by deteriorating doctor-patient relationship has also been reported amongst general practitioners in 10 E.U. states (Bensing et al., 2013). In order to make sense of the deterioration in their relationship, it is necessary to look at the workplaces these doctors and nurses operate in to provide care to patients.

This study attempts to examine the following question: How does the healthcare workplace in West Bengal, India, work to foster or diminish the doctor-nurse-patient relationship? First, I hypothesize that healthcare workplace conditions foster the deterioration of the doctor-nurse-patient relationship in contemporary West Bengal. These working conditions result from structural factors (hospital environment, healthcare management, public health policy) rather than inherent interpersonal conflict. These structural factors shape the habitus of the actors involved—doctors, patients and nurses and promotes the deterioration of the doctor/nurse-patient relationship.

In order to test my hypothesis, I employ Bourdieu's notion of habitus and social space. I categorize the actors within this setting into three classes—doctor, nurse, and patient, and attempt to construct the class habitus for each of these agents. I characterize the social spaces these agents inhabit and construct their habitus utilizing a combination of secondary source data, qualitative respondent narratives, and quantitative respondent data to develop a social theory driven understanding that reveals the causes of the deteriorating doctor-nurse-patient relationship. Through an understanding of some of the workplace interactions and attitudes, we can perhaps understand the shifts in attitudes toward doctors as respected members of society to targets deserving of physical, verbal, and emotional violence. This theoretical approach will help elucidate the contradictory attitude toward doctors as deserving both praise and contempt.

## Methods

### Empirical data collection

#### Conceptualization of semi-structured interview schedules

The semi-structured interview schedules containing both closed and open ended questions was designed to capture demographic information of the respondents, and to gain an understanding of their experience of the hospital as a workplace (doctors and nurses) and as a care facility (patients), and their relationships amongst each other. The final interview schedules utilized have been provided within Supplementary material 1—Semi-structured Interview Schedules.

#### In-person in-depth semi-structured interviews

**Figure 1:**
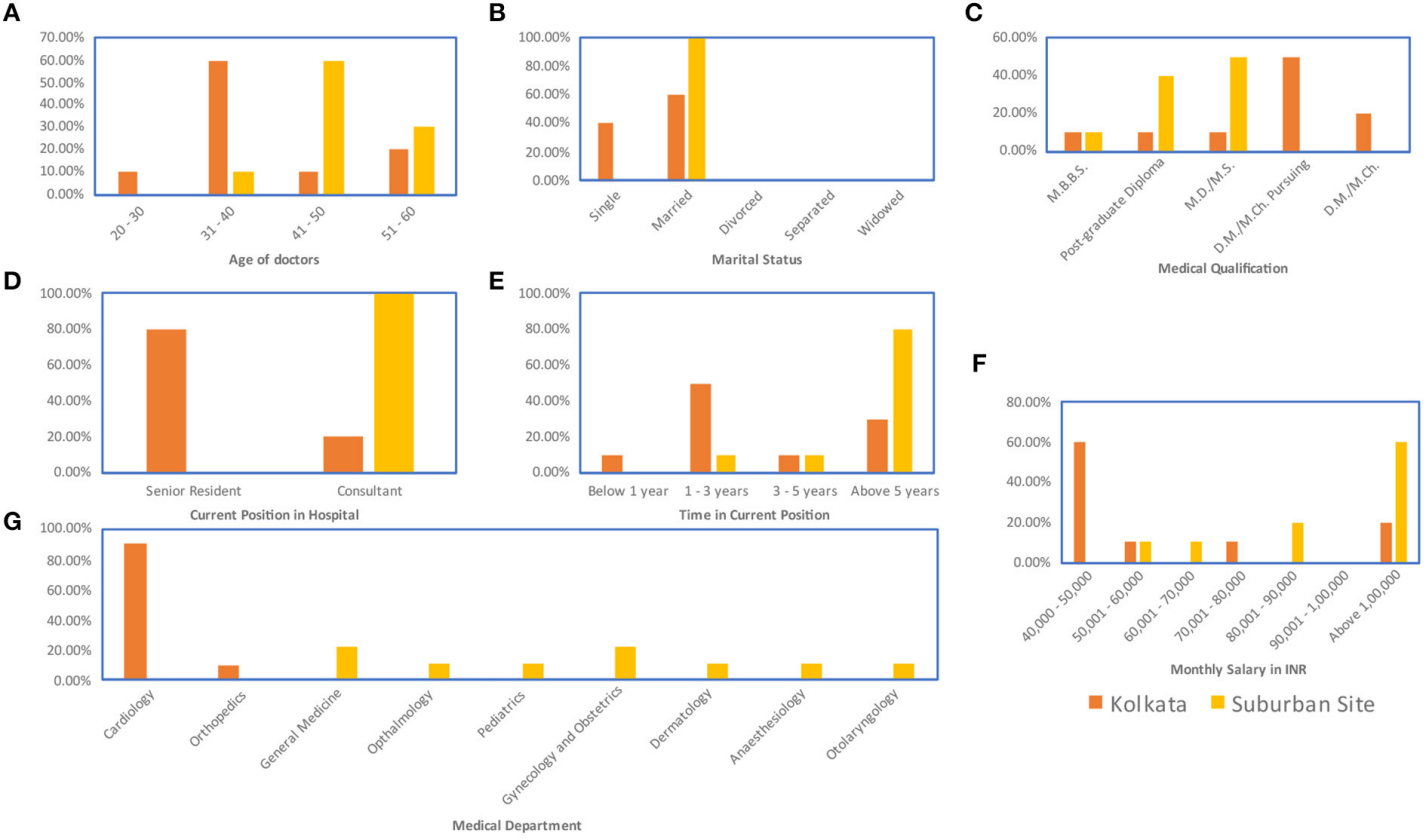
Demographic data for doctor respondents: **(A)** Age **(B)** Marital status **(C)** Medical qualification. Within the Indian system, medical education is provided at a bachelor's degree stage, therefore the primary qualification of a practicing doctor in India is Medicine Bachelors and Bachelors in Science (M.B.B.S), doctors obtaining an optional pre-master's degree specialization receive a post-graduate diploma, doctors obtaining a master's degree specialization in either medicine or surgery receive an M.D./M.S., and doctors obtaining a doctorate degree specialization receive an M.D./M.Ch. **(D)** Current position in the hospital **(E)** Time in current position **(F)** Monthly salary in INR **(G)** Medical department.

**Figure 2:**
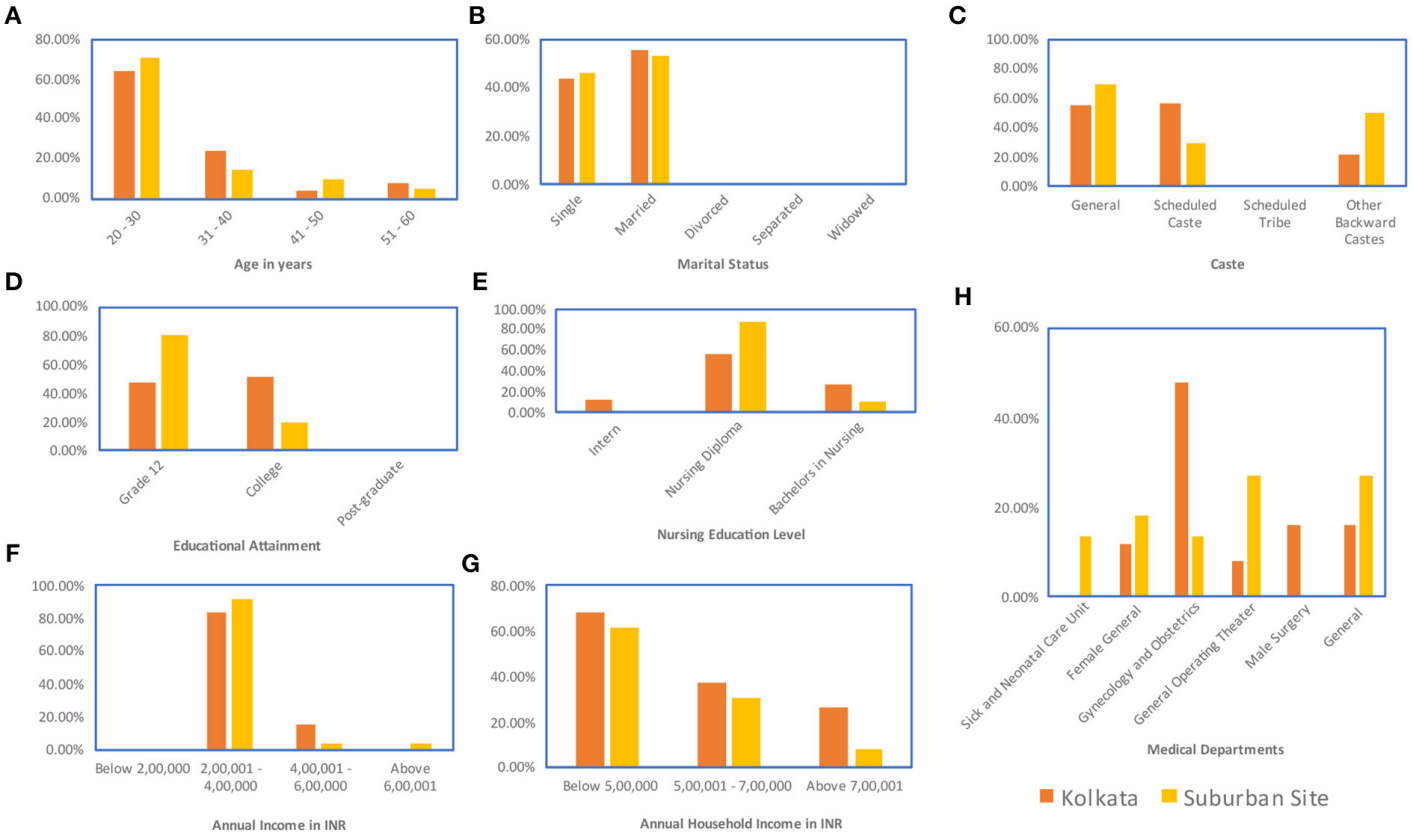
Demographic data for nurse respondents: **(A)** Age **(B)** Marital status **(C)** Caste **(D)** Educational attainment **(E)** Nursing education pursued—within the Indian system, trainee nurses can work as interns in the hospital during their nursing training period, a two-year program is available for nurses to get their diploma in nursing, some nurses chose to do this after they receive an undergraduate bachelor's degree in another field, and a four-year undergraduate bachelor's degree in nursing is also available for nurses **(F)** Annual income **(G)** Annual household income **(H)** Medical department.

**Figure 3:**
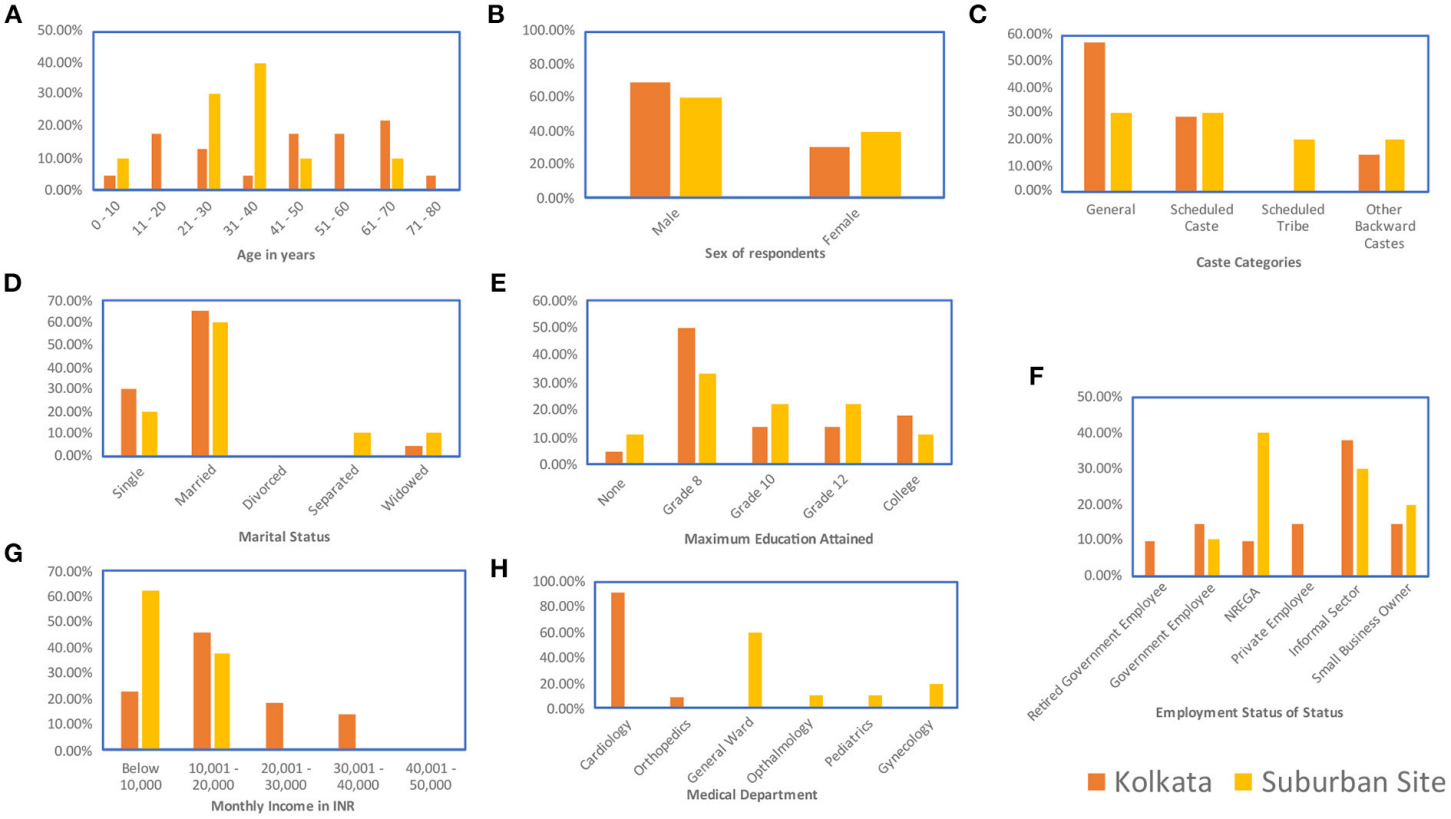
Demographic data for patient respondents: **(A)** Age **(B)** Sex **(C)** Caste **(D)** Marital Status **(E)** Maximum level of educational attainment **(F)** Employment status, including patients employed under a 100 day right-to-work employment guarantee provided by the Indian Government under the National Rural Employment Guarantee Act (NREGA), for pediatric patients, this panel captures the employment status of the head of household **(G)** Monthly income in INR **(H)** Medical department within which the patient was seen.

Primary empirical data was collected through in-person, in-depth, semi-structured interviews schedules with both open and closed-ended questions conducted throughout 2018 across 5 hospitals in Kolkata (major metropolitan center, population 15 million) and one hospital in a suburban area with population 100,000. These hospitals contained a mix of secondary and tertiary care facilities. The respondents consisted of 51 nurses, 20 doctors, and 33 patients recruited using purposive and snowball sampling. Nurse and doctor respondents were evenly distributed across both sites; 23 out of 33 patients were interviewed at the Kolkata sites. Respondent demographics have been provided in Figures 1–3.

Separate interview schedules were drafted for nurse, doctor, and patient interviews. Questions were posed in Bengali to patient respondents, and answers were translated into English *in situ* and noted down. Doctor and nurse interviews were conducted in a mixture of English and Bengali and the responses translated *in situ* and noted down. Interviews were not recorded due to participants' hesitance to be recorded. Nurse and doctor interviews were conducted within the hospital that respondents were affiliated to. Patients were interviewed outside of the doctor's office after their consultation with the referring doctor. Since government hospitals may have many individuals claiming to be patients to researchers or journalists, referrals from doctors were used to identify bona fide patients.

These data have received IRB permission from the University of Chicago (IRB21-0507).

### Utilization of a theoretical framework–Bourdieu's social theory

Responses to closed-ended questions were exported into a spreadsheet format. Responses to open-ended questions were qualitatively reviewed. In order to use these analytical methods, a brief economic, political, and historical analysis was conducted. Data were interpreted using the analytic concepts of forms of capital, social space, and habitus. A social space analysis was performed using data from the interviews along with the economic, political, and historical background information. In attempting to understand the methods by which the respondent groups were classifying their medical interactions and experience, the responses to the semi-structured interview schedule were initially analyzed and a respondent group habitus for each group (doctor, patient, and nurse) was constructed. For nurses and patients, this analytical framework led to the construction of a single habitus for each group. The responses to the doctor group indicated a disharmony between their desire to provide comprehensive care and the limitations imposed on them by the wider West Bengal healthcare system. Analytically, this disharmony was resolved by the construction of two habitus, each representing an extreme—the first is grounded in the idea of the doctor as a benevolent healer, and the second is grounded in the doctor as an agent of the West Bengal healthcare system.

### Economic, political, and historical analysis

Primary sources (Government circulars, WHO and OECD reports), press reports, and academic publications have been utilized for budgetary figures and analysis. Secondary news sources detailing the election politics in West Bengal were utilized to provide context to understand the relevant political milieu. Secondary sources were utilized to construct a genealogy of the West Bengal healthcare system and the role of doctors, patients, and nurses within this system. Secondary sources were utilized to provide context to understand Kolkata's role in acting as a gateway to modern medicine into India, the Bhore report as a framework for the three-tiered organization for the West Bengal healthcare system, and Dr. Bidhan Chandra Roy's role in framing Bengali conceptualization of the role of a doctor.

## Analytical concepts utilized in the study

### Bourdieu's forms of capital

Bourdieu's discussion on the forms of capital begins by defining capital as accumulated labor in an embodied form that has been appropriated—an obvious concept when considering economic capital, but a novel concept for social and cultural capital (Bourdieu, 1986, p. 241).

Cultural capital is accumulated appropriated labor that exists in the form of a person's qualifications, natural and acquired abilities, and knowledge. It exists in three forms—embodied state, objectified state, and institutionalized state. Embodied cultural capital mostly resides within and in relation to the body of its holder and it ceases to exist with the perishing of its bearer. It can be covertly transmitted through hereditary means. Objectified cultural capital resides in material objects that have been imbued with cultural meaning. This form of cultural capital is more openly transmissible through the transfer of the object itself. Institutionalized cultural capital resides in the socially recognized qualifications given to the holders of this capital. The institution makes the embodied cultural capital more recognizable and transmissible (Bourdieu, 1986, p. 243–47).

Social capital is accumulated appropriated labor that exists in the form of a person's social networks, or their membership in groups. The size of the social network determines the volume of the social capital possessed by an individual. An individual's social capital is magnified by their economic capital, and the economic capital of those in their network. The network of relationships is produced through the investments of time, emotion, and labor, which reproduce social relationships that are usable short and long term (Bourdieu, 1986, p. 247–50).

In this study, the forms of capital held by each of the agent classes is analyzed to generate a qualitative understanding of the operative social spaces to enable the construction of the habitus of each of the agent classes.

### Habitus

Habitus is the incorporation of the social world into the body from which follow schemes of perception, cognition, language and more (Bourdieu, 1984, p. 170; Bourdieu, 2000, p. 138–39). It is the presence of the past in the present, in essence, it is a history that guides the dispositions and preferences of the individual (Bourdieu, 2000, p. 210–12). Dispositions and preferences could be considered as methods by which an agent classifies new observations and patterns (Bourdieu, 1984, p. 6). A habitus is a structured structure that enables the individual to classify objects and events around them. A habitus is also a structuring structure that enables the individual to build upon a classification system that they already possess and utilize. The structuring structure is also referred to as the generative principle of the habitus—the principle by which the individual with their habitus classifies the world and further generates their habitus (Bourdieu, 1984, p. 170).

In order to understand and account for the practices of an individual belonging to a class (class condition), the habitus that lead to those practices needs to be constructed. This process begins with the construction of an objective class, the set of agents within similar conditions of existence, having similar conditionings, similar systems of classifications and possessing similar properties (Bourdieu, 1984, p. 101). The practices of this class can then be dissected to understand the habitus directing the actions taken by the class. Finally, habitus do not occur in isolation but within a given social space (Bourdieu, 2000, p. 134–35; Reed-Danahay, 2020, p. 30). Therefore, to construct the habitus, it is necessary to understand the social spaces within which these habitus operate to generate practices.

### Social space

Understanding the conditions of existence, conditionings, system of classifications, and properties of an agent is akin to understanding the total capital possessed by the agent, and the nature of the distribution of this capital. Bourdieu writes about the construction of this space occupied by the agents in three dimensions. The first dimension of this space captures the total capital possessed by the agent, the second dimension captures the distribution of this capital in the three forms, and the third captures the evolution of the first two spaces through time (Bourdieu, 1984, p. 114). While molded by history, this analysis does not consider the temporal evolution of the relative positions of the agents within the two dimensional space representing the total capital possessed and the relative weights of the forms of the capital possessed (Bourdieu, 1990b, p. 128). Within this two-dimensional space, it is necessary to consider not just the modal individual from the agent class, but also the capital possessed by the agent class as a whole (Bourdieu, 1989, p. 17). While a quantitative social space analysis is beyond the scope of this work, a qualitative social space analysis can help generate the backdrop for the construction of the habitus of the agents in this study.

## Results

### Health infrastructure, health policy, and health politics of West Bengal

West Bengal has a three-tiered healthcare system. There is a primary healthcare network focused on small clinics, a secondary system comprising of district and sub-divisional hospitals, and a tertiary system providing “specialty and super specialty care”. Primary care is provided by 11,613 facilities, of which 10,356 are outpatient care only, with provisions for 16,498 hospital beds; secondary care is provided by 126 facilities with provisions for 29,508 hospital beds; and tertiary care is provided by 12 facilities with 12,641 beds. Of the 107,346 hospital beds in West Bengal, 55% are operated by the State Health Ministry and approximately 32% are operated by private entities (Government of West Bengal, 2010).

Noting the number of hospital beds—107,346, and West Bengal's population in 2011 (similar time-frame as the hospital beds data)—91,276,115, the number of beds per 1000 population is 1.17, which is higher than India (0.5) and half the number as the UK (2.5) (OECD, 2019). Per capita funding of healthcare in West Bengal, despite recent increases is $13.44 (based on Feb 27, 2021 exchange rates) or $44.97 based on Purchasing Power Parity adjustment (OECD, 2012). Therefore, despite the availability of beds, healthcare remains under-funded. The hospitals and healthcare facilities are inadequately staffed – doctor to population ratio was 1:10411 (Bose, 2019), about thirty times lower than that of UK 1:355 (WHO, 2021). Statistics regarding the number of hospital beds and doctors cover both private and public healthcare systems. A majority of Indian healthcare expenditures occur out-of-pocket and costs of outpatient and inpatient care are inflating at 15 and 31% annually (Prinja et al., 2012). Government spending on healthcare as of 2012 is 0.9% of GDP, while total spending on healthcare is 5% of GDP (Prinja et al., 2012). The difference between public spending on healthcare, and total spending on healthcare makes it clear that the Government-run hospital system is not adequate to handle its estimated patient load.

West Bengal's healthcare system has not coped with the population or economic growth of the state. I argue that West Bengal Government attention to healthcare is driven primarily by electoral exigency rather than responding to actual healthcare need. Major elections were held in West Bengal in 2016 (Arvind and Ahamed, 2016), 2019 (Ministry of Statistics and Programme Implementation, 2020) and in 2021 (Beauchamp, 2021). Elections for the State parliament were held in 2016 which was a test for the current ruling party which overthrew 34 years of continuous state-level communist rule in 2011 (Barman, 2011; Arvind and Ahamed, 2016). The current ruling party also then won a hard-fought election in 2016. The elections in 2019 were for the seats of West Bengal in India's national parliament, the Lok Sabha, and the current ruling party was fighting off well-funded opposition parties, who ultimately reduced the share of the current ruling party seats in the Lok Sabha from 34 to 18 out of 42 seats for West Bengal. Finally, the ruling party won a third term in a hard-fought election as a majority in the State government in 2021. Budget forecasts for the state are generated in January, and thus looking at the forecasts for the 2015–2016 year, we observe a 14% increase in per-capita healthcare expenditure (Bose, 2019), followed by <5% increase, and even 2% decrease announced in January 2018. However, in January 2019, the West Bengal chief minister announced a 8.1% year-on-year increase in healthcare expenditure (Census, 2011; Government of West Bengal, 2019) followed by a 31.3% increase in January 2021 (Census, 2011; Government of West Bengal, 2020). The timings of these increases acutely reflect a few political events. In 2016, the current ruling party required the votes of the rural population and minority population, many of whom use public healthcare services (Bose and Dutta, 2014; Gupta, 2016). In 2020, it appears that the current ruling party had to respond to the doctor's strike following the assault of a doctor in Kolkata's NRS hospital (Basu, 2019a) and the loss of votes in the national election. Finally, in 2021, it would appear that the current ruling party had to solidify its position to fend off opposition parties encouraged in 2019, and strong anti-incumbency sentiments (Beauchamp, 2021; PTI, 2021a). Therefore, it appears that the healthcare budget of West Bengal is deeply tied to its politics.

Poor attention, political exigency, and cynicism in the administration of healthcare policy in West Bengal is neither new nor recent, as even during the extended communist rule of West Bengal from 1977 to 2011, government attention to healthcare was criticized:

“*Of course, no government in its right mind would think that they would improve the health care system in the state.”*

“*In its long uninterrupted innings of 27 years, this is not the first time that the Left Front-run government in West Bengal is faced with such embarrassment on the health front.”* (Chakraborty and Mukherjee, 2003).

Thus, understanding healthcare, especially state-provided care in West Bengal, involves understanding some of the politics in West Bengal. Elections in West Bengal are characterized by remarkable violence compared to other Indian States (Mukherjee, 2020).

In a milieu in which bureaucratic documents reveal the politics of healthcare budgeting, and the political institutions are infused with a history of violence, it can be expected that confrontations with the healthcare system mutate into political confrontations and are thus imbued with violence.

### Historical status of doctors and government hospitals in West Bengal

The capital city of West Bengal—Kolkata, erstwhile called Calcutta occupies a unique space in the history of the colonization of India. Kolkata was set up as a port by the English East India Company in 1690 and served as a focal point for the networks of knowledge and trade connecting colonial India to the world (Raj, 2011). The first plans for the construction of a medical college in Kolkata were proposed in 1828, and eventually, the construction of India's first modern medical school and college—the Calcutta Medical College started in 1835. The new Calcutta Medical College faced significant scrutiny and apprehension from traditional medical practitioners (offering services using Ayurveda or Unani methods of medicine) through the 1800s (Bhattacharya, 2014). In time, as India expanded, further hospitals were built to accommodate her growing population. The next marker in the establishment of a healthcare system was the Bhore report commissioned in 1946, just prior to India's independence in 1947 (Ma and Sood, 2008). This report recommended a three-tiered healthcare system to provide care to both urban and rural areas of the country. The three-tiered structure established by the Bhore report in 1946 is evident in the current layout of the public health infrastructure in West Bengal today.

One of the key figures in post-Independence West Bengal was Dr. Bidhan Chandra Roy (BC Roy). He was a medical doctor, statesman, and administrator who was the Chief Minister of West Bengal from 1948 to 1962 and led the State through its prosperous years. He established the Indian Medical Association in 1928 and the Medical Council of India—arguably initiating doctor advocacy in India (Chatterjee, 2019). Within Kolkata, he established 7 hospitals. Within West Bengal he established five new cities. His birthday, July 1st is celebrated as Doctor's Day in India. It is within this context that many of the contemporary doctors see their profession and themselves. Dr. BC Roy's influence on modern West Bengal perhaps has cast its net and led to a perceived exalted status of all doctors in West Bengal. The doctors interviewed also reported similar exalted perception to demonstrate the contrast in attitude observed in the deteriorating perception of doctors in contemporary times.

“*False hype of doctors being God.”*—Dr. S. Roy.

“*Because common people think that sometimes doctors can do everything. It's all in their hands.”*—Dr. J. Chatterjee.

In these responses, we observe the doctors as being aware of their perceived omnipotence and their reluctance to take on such a powerful paternalistic role in the medical establishment. Understanding and characterizing the habitus of the doctors and patients in this setup could illuminate the barriers to a cooperative shared decision making based doctor-patient relationship (Spinnewijn et al., 2020). The characterization of the habitus requires three components—an understanding of the forms of capital as postulated by Bourdieu, an analysis of the social space within which the West Bengal Government Hospitals operate, and an analysis of the social space relationships between the doctors, patients, and nurses.

### Respondent demographics

Respondent demographics have been provided in Figures 1–3.

**Figure 4:**
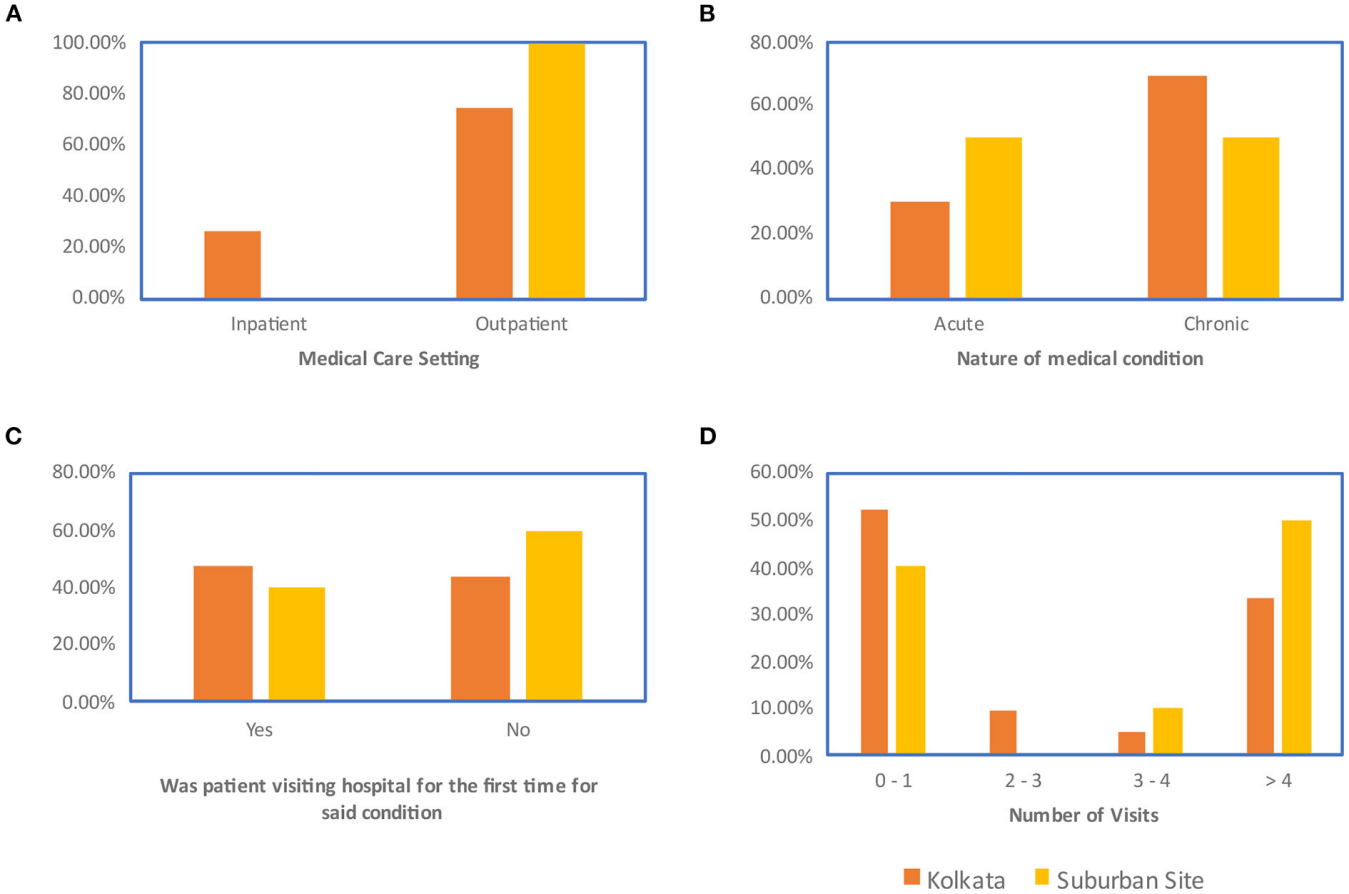
Nature of patients' medical visit at the time of interviewing: **(A)** Medical care setting **(B)** Nature of the medical condition **(C)** Whether the visit represented a first-time visit for a particular ailment **(D)** Number of hospital visits to address the particular ailment.

Data regarding the nature of medical issue that brought the patients to the hospital on the day of the interview have been provided in Figure 4.

### Social space analysis of West Bengal government hospitals

“*Thus, agents are distributed in the overall social space, in the first dimension in accordance with the overall volume of the capital that they possess in different kinds and, in the second dimension, in accordance with the structure of their capital, that is, in accordance with the relative weight of the different kinds of capital, economic and cultural, in the total volume of their capital.”*—(Bourdieu, 1990b, p. 128).

In order to understand the interactions within Government hospitals, it is necessary to perform a preliminary analysis of the social space occupied by Government hospitals within the secondary and tertiary care infrastructure in West Bengal. In terms of economic capital, private tertiary hospitals are expensive and well-funded and private secondary hospitals are also comparatively well-funded compared to their government run counterparts. Recent sales of a single private tertiary care hospital in Kolkata valued it at US $130 million (Balakrishnan, 2018), about 10% of the total West Bengal Government Budget which funds 138 secondary and tertiary care hospitals. Therefore, with regards to economic capital, private hospitals stand far apart from comparable Government hospitals.

“*More precisely, capital exists and acts as a symbolic capital … in its relationship with a habitus predisposed to perceive it as a sign, and as a sign of importance, that is, to know and recognize it on the basis of cognitive structures able and inclined to grant it recognition because they are attuned to what it is”*—(Bourdieu, 2000, p. 242).

Due to their high economic capital, the privately-run hospitals can afford to hire doctors with credentials from the global North. These credentials, typically from the USA, UK, Canada, or Australia have higher symbolic capital than equivalent certifications from Indian institutions that can often be much more competitive to obtain. While a formal analysis of the composition of the symbolic capital of hospitals in West Bengal is beyond the scope of this study, an indicator of the conversion of economic capital into cultural capital performed by private hospitals can be seen in the manner in which qualifications of doctors are listed on private hospital websites. In the website for AMRI (AMRI, 2020), a renowned private tertiary care hospital in Kolkata, foreign credentials are clearly highlighted in Dr. Amit Roy's write-up, while there is no explicit mention of institutions of credentials in Dr. Amitabha Roy Choudhury's writeup. This highlights the possession of relatively less valued Indian credentials. Additionally, the National Board of Examinations of India does not require additional licensure examinations from medical graduates from US, UK, Canada, Australia and New Zealand, but explicitly requires it for graduates from all other countries, including India (Sharma, 2015). In Pascalian Meditations, Bourdieu addresses the nature of all capital to become symbolic capital, and therefore nudges analysis toward the symbolic effects of capital (Bourdieu, 2000, 240–45). In the preferential treatment of education credentials (cultural capital) from certain developed countries, and the exclusive advertising of the foreign credentials of some of its physicians, the private hospitals and wider society exhibit recognition of certain credentials as being superior to Indian equivalents. Thus, in Bourdieu-ian terms it can be deduced that the private tertiary care hospitals attribute symbolic investiture to foreign credentials in order to demonstrate superiority to attract the economic capital of affluent patients. Both government and private hospital specialists pass the same advanced licensure examinations, and they are at par with each other in terms of medical expertise and skills. However, a majority of patients in West Bengal cannot afford healthcare from doctors possessing these perceived higher qualifications and thus receive care in a government hospital. Over 80% of the hospitalizations in West Bengal occurred in a Government hospital (Almeida et al., 2017). Even though private tertiary hospitals serve patient populations with higher individual social capital, the Government hospitals by virtue of their higher patient numbers can compete in terms of social capital. Additionally, private hospitals tend to invite political anger (Thakur, 2020) from the ruling government, while Government hospitals are advertised as sites of health policy progress (PTI, 2021b). Private hospitals have also been subject to legislation to protect patients from medical negligence and over-billing, while Government hospitals are exempt from this legislation indicating political favor supporting Government hospitals over private hospitals (Telegraph Bureau, 2017).

Based on these preliminary analyses, private hospitals are situated apart from Government hospitals in the first dimension of social space when considering total capital possessed. In the second dimension of social space, considering the structure of the possessed capital other differences are observed. In terms of economic or symbolic capital, the private hospitals stand apart from Government hospitals. The hospitals are closer together in terms of social capital. While private hospitals accrue their social capital from small numbers of patients having large individual economic and cultural capital, the Government hospitals accrue their social capital from large numbers of patients having low individual economic and cultural capital and from ruling party-political favor. This study focused on the Government hospitals because their patient population is more representative of the general population.

### Social space analysis of doctors, nurses, and patients

The doctors, nurses, and patients have different amounts of total capital, and their capital is structured differently as well. Considering each of these groups in terms of the capital they possess based on the data collected can elucidate this relational space and make understanding the interaction of their habitus more comprehensible. Considering economic, cultural, and social capital in turn.

By virtue of their training, senior doctors have the highest amount of economic capital amongst the three groups. Doctors interviewed in Kolkata were mostly junior doctors [<5 years of experience, 60% of the respondents, Figure 1E] while those interviewed at the suburban site were mostly senior doctors (>5 years, 80% of respondents, Figure 1E). The lowest salary scale for the junior doctors was ₹40,000 to ₹50,000 per month (Figure 1F), which was low given the educational qualifications of the doctors but high compared to the other groups in the analysis. More experienced doctors earned at least ₹1,00,000 per month (Figure 1F). The median income for the nurses across both sites was closer to ₹25,000 per month (Figure 2F). For patients, the average income across both sites was ₹15,000 per month (Figure 2G). Therefore, while the lowest paid doctors and the highest paid nurses had similar incomes, the doctor income was higher than the nurse income and the nurse income was a little higher than the income of the patients. So, in the social space, in terms of economic capital, the doctors are far apart from the nurses and the patients, who are closer together.

In considering cultural capital of the three groups, it is necessary to consider cultural capital as being accrued from three different sources—familial background representing embodied cultural capital, educational attainment representing institutionalized cultural capital and professional and occupational status, which may be considered as another embodied form of cultural capital. Considering familial background, about 50% of the doctors reported having another doctor in the immediate family.

Data were collected on the caste backgrounds of the respondents. The categories of caste were General caste, which covered those castes that are not positively discriminated by Indian law. The other categories were Scheduled Caste, Scheduled Tribe, and Other Backward Class categories (ST, SC, and OBC) which indicate castes that are positively discriminated by Indian law. In Indian law, positive discrimination implies that for government jobs and educational seats spots are reserved for ST, SC, and OBC candidates and cannot be filled by a person of a General caste. This policy was enacted to recognize the systemic oppression of these castes throughout Indian history (Basavaraju, 2009; Vishwanath, 2021). The doctors were all from General caste. The doctors interviewed also skewed male, with only one female respondent out of 20. Approximately 54% of the patients interviewed belonged to ST, SC, or OBC categories (Figure 3C). Female respondents were better represented in the patient respondent set (33.33%, Figure 3B). For the nurses, all respondents interviewed were female and approximately 38% belonged to ST, SC, or OBC categories (Figure 2C). Therefore, on non-educational parameters, doctors are located apart from the nurses and the patients both in terms of familial background and in terms of caste background compared to the nurses and patients who are located closer together in this respect.

Considering the educational component of cultural capital, the doctors, nurses, and patients are located far apart from each other. All the doctor respondents have at least an MBBS (5-year undergraduate medical curriculum in India) with over 90% pursuing further accredited medical education (Figure 1C). Therefore, the doctor respondents all have over 17 years of formal education. All the nurse respondents had graduated high school, and about 36% had a graduate degree (3-year program) with some having a nursing diploma (1 year program) (Figures 2D,E). Some of the nurses interviewed were also pursuing other specialized nursing training programs (<1 year) (Figure 2E). Therefore, the nurse respondents all had between 12 and 16 years of formal education. However, for the patient respondents, over 80% had less than a high-school education, including about 6.45% of the patients interviewed who reported being illiterate (Figure 3E). Therefore, in terms of both economic capital, and non-education-based cultural capital, while the nurses had been in a similar space as the patient population, the higher education of the nurses implies that they have significantly higher cultural capital than the patients and are thus set apart.

The nature of the job sets the cultural capital of the nurses apart from that of the patients. A majority of the patients, nearly 55% work in the informal sector or as wage laborers (Figure 3F). Neither of these forms of employment are considered a pathway to the middle class in India. However, the nurses consider their job to be a career and a pathway to the burgeoning middle class of India. The nurses also reported thinking of their job as a way to gain financial independence and consequent empowerment, and they contributed at least 40% to their family income (Figures 2F,G). There is perhaps an additional layer of cultural capital attached to the empowerment that the nurses derive from their jobs.

The social capital of the three groups is challenging to assess. One approach is to look at it at two scales, at the scale of an individual and at the scale of a group. An individual doctor perhaps has the highest social capital. Due to the nature of the profession, their social networks are large and consist of other doctors, nurses, pharmacists, and medical supply sales representatives in addition to their non-professional social networks. Many members of their social network have comparatively higher economic capital, which amplifies their own social capital. An individual nurse has a comparatively smaller amount of social capital, and the individual patient has the least amount of social capital. Thus, in the relational hierarchy of social capital, patients are at the lowest rung, while nurses are higher, and doctors are at the highest point with respect to individual social capital. However, as a group, different dynamics take place.

The doctor lobby, a reflection of the social capital of the group of all doctors is fairly strong, and can get the Government and public attention when it chooses to Basu (2019a). However, patients outnumber doctors 10411:1 (Bose, 2019), therefore, even if the individual patient social capital is low, the sum of many small social capitals can often equal or exceed the sum of few large social capitals. Additionally, due to the intertwining of healthcare and politics, the larger patient population and its power at the ballot box influences politicians, further boosting the social capital of the patient population as a group. The inclusion of larger number of patients as part of the State-run health insurance scheme in West Bengal without appropriately matched hiring of doctors serves as a demonstration of the social capital of patient population triumphing in a battle over the social capital of the doctors (Chakraborty, 2020; Government of West Bengal, 2020). The nurses as a group do not have the numbers to compete with the doctors and the patients in this respect.

Considering the two dimensions of social space, total capital, and structure of the capital, it is clear that the doctors, patients, and nurses occupy different relational spaces. Due to the outsize influence of social capital of the patients as a collective, it is hard to adequately understand if the total capital of the doctors exceeds that of the patients. However, in terms of the second dimension or the structure of the capital, while doctors hold proportionally high amounts of all three forms of capital, and nurses hold proportionally lower amounts of all three forms of capital, patients disproportionally hold social capital while possessing comparatively lower amounts of cultural or economic capital. Due to the disproportional distribution of patient population social capital, in case of a conflict, the ingrained disposition of patient population is to deploy their social capital to resolve a dispute. This deployment may often look like mob attacks, coordinated smear campaigns or crowdsourced knowledge being used to attack the doctors' and nurses' cultural capital. Understanding patient, doctor, and nurse perspectives on medical encounters imbued with an idea of the operative social space can help highlight how their individual habitus intersects with their field of practice to either engender conflict or mitigate it.

### Interpreting respondent reflections on medical encounters

#### Doctor respondents

Doctor respondents in both Kolkata and the suburban site (65% of doctors interviewed) acknowledged that the traditional paternalistic model of doctor-patient relationship is not their typical experience. They reported that their interactions with patients led them to perceive that the patients had lost their faith in doctors and distrusted them. A doctor respondent at the suburban site (a secondary hospital) often felt that the families of patients became aggressive whenever treatment outcomes were unsatisfactory even if realistic and expected. The doctor respondents at all the sites reported feeling an omnipresent threat of physical violence in field interviews. Another suburban doctor has responded to this omnipresent threat by recommending conservative treatment plans and refraining from taking risks with critical patients or patients with the slightest complication and opting instead to refer them to the nearest tertiary care center. A priori, doctors at a secondary care center choosing to be liberal in referring patients to a tertiary care center may seem like a preferred strategy but is likely to crowd the tertiary center. Transferring to a tertiary care center increases patient and dependent's hardship, as, in the suburban setting, the tertiary center was 60 km away, and would add travel and transfer time for patients, many of whom may have already spent considerable amount of time to get to the secondary care center. Moreover, I would hypothesize that over a longer time, this liberal referral policy would lead to a loss of experience and skill dealing with more complex cases in the secondary care setting.

Most doctor respondents in both places maintained (65% of doctors interviewed) that there has been a shift from paternalistic model, but this shift had not yet paved the way for a collaborative model between doctor and patient. This can be attributed to the low educational background of the patients, only 20% of whom had graduated high school, and over 6% of whom were functionally illiterate (Figure 3E). Traditionally, this gap in educational attainment is likely to be correlated with higher dependence on doctors for any decision related to their ailment. In total 80% of doctor respondents from both metropolitan and suburban sites stated that their communication patterns differed with differing socio-economic backgrounds of patients, 20% said it remained the same with all patients. Doctors reported that they believed that patients have an immense role in getting cured. They believed that it was necessary for patients to have faith and reliability in doctors, for patients to be compliant, follow advice, comprehend the gravity of their disease, and have patience in the treatment procedure. They desired patients who explained their problems explicitly without hiding anything from them, had a positive frame of mind, and updated and reported to them immediately about any unexpected effects during treatment.

“*two groups of patients. One group of patients who are illiterate, they trust more. Another group of patients who are educated trust less.”*—Dr. A. Sarkar.

“*dual behavior. Less acceptance of unsuccessful treatment.”*—Dr. R. Ganguly.

“*they should know about the risk and treatment modalities of the setup in which they are coming.”*—Dr. P. Ghosh.

“*patients should have minimum knowledge. Ignorance is not an excuse; else you will get exploited.”*—Dr. M. Dutta.

When asked to speculate about the reasons for medicine no longer being regarded as a noble profession meant to save lives and the reasons why they are at the center of a “blame culture”, 55% of doctor respondents argued that they are victims of the patient overload in government setups, they are overburdened by work, local news media acts as a rabble-rouser with their journalism that does not appreciate the complexity of medicine, and the general disbelief of society. At the State-level, the unideal doctor-patient ratio is an indicator of the patient overload. However, the data collected reveal a more specific picture of this overload. Amongst the doctor in Kolkata, 40% of the doctors interacted with more than 150 patients per day in the Outpatient Department (OPD), 30% interacted with more than 100 patients and 10% said they deal with more than 200 patients per day. A doctor interviewed at one site in Kolkata stated that he visited not more than 20 patients a day. This was only because this site is a Government-aided hospital and the doctor had the autonomy to regulate and decide the number of patients they want to treat. The numbers are similarly acute at the suburban site as well—40% of doctors treated more than 150 patients in OPD. For inpatient departments (patients admitted to the hospital), 50% doctors see approximately 50 patients per day in Kolkata and 70% doctors at the suburban site attend to 20–25 patients per day. In Kolkata, 30% doctors can give 2–5 min to patients in OPD, 30% can give 10 min and 20% doctors reported that the time dedicated varied and could increase depending on the type of illness. A similar picture was obtained at the suburban site, 50% doctors give 2–5 min, 20% give less than 2 min, 20% said time depends on illness, and one of the anesthesiologist respondents reported that time given to patients will depend on length of operation. Therefore, crushing patient burdens are part of the problem, and can be understood at both the scale of individual hospitals and the health system overall. Some of the doctor's comments laid out the mechanism by which faulty journalism and social disbelief contribute to their declining reputation.

“*Negative prochaar beshi. One bad case gets highlighted. 3rd party- media. Political parties, unknown people creating fuss.”*—Dr. M. Kundu.

“*Because patients are getting more knowledgeable. They can understand minor errors. Now patients have more information. But that is a generalized information. A particular disease has different symptoms for different people. But people miss this point. News channels are also responsible. People get instigated. Bad reporting. Huge knowledge gaps. Gaps in basic knowledge. Wrong technical terms used.”*—Dr. R. Sheikh.

“*Lack of communication training in medical curriculum; Inadequately trained media to cover medical subjects. False hopes regarding health-related diseases done by Pvt. organizations.”*—Dr. L. Gupta.

“*Because common people think that sometimes doctors can do everything. It's all in their hands. They misunderstand that doctors intentionally harm patients. Media unfairly projecting doctors. Media is politically influenced. According to politicians every treatment should be free.”*—Dr. N. Chakraborty.

In order to comprehend the mechanisms driving the deterioration of the reputation of doctors, I started from trying to understand how doctors see their own role in the deterioration of the doctor-patient relationship. An overwhelming majority (90%) of the doctors reported that “thinking that the physician will always know the exact diagnosis at first consultation and start treatment immediately” is a cause for patient dissatisfaction. This alludes to the patients having an exalted perception of doctors and their ability to provide diagnosis and treatment. However, when asking doctor respondents about their patient loads, 90% of the doctors reported seeing over 100 patients within an outpatient setting during 8–12 h shift and reported spending between 2–10 min per patient within the same setting. Therefore, while there is this perception that doctors should be able to diagnose and treat immediately, there are real-world pragmatic considerations that preclude this. Thus, to me, a single habitus could not fully account for the doctors' responses. Two habitus, each grounded in an extreme—idealized doctor vis-à-vis cog in an overburdened healthcare system, could help account for the doctors' responses.

The first habitus is structured by the historical and idealized status of doctors and the second habitus is structured by the working conditions currently faced. The first habitus is likely to be more compatible with either a purely paternalistic relationship or a truly equal collaborative relationship. The second habitus is likely to be more compatible with the practice of medicine within the West Bengal Government hospital social space and the doctor, nurse, and patient social space. Understanding the interaction of these habitus with the social space landscape may reveal how the gap between these two habitus is leading to a deteriorating doctor-patient relationship.

“*The habitus is not only a structuring structure, which organizes practices and the perception of practices, but also a structured structure: the principle of division into logical classes which organizes the perception of the social world is itself the product of internalization of the division into social classes.”*—(Bourdieu, 1984, p. 171).

Understanding a habitus requires understanding both its current structure as well as the generative schemes ingrained in that structure that leads to its growth. The first habitus structures the self-image of the doctor as a benevolent healer, one who takes the time to both understand and address the physiological ailments and associated emotions of the patients. This habitus ingrains the idea of a doctor as being an upstanding citizen performing an essential and valued vocation within society. It inculcates the idea of the doctor as being omnipotent and powerful enough to address the illnesses of the society that they are a part of. The generative schemes of this habitus derive from the Hippocratic oath in a general sense, and for doctors within the West Bengal Government Hospital system, the outsize legacy of Dr. Bidhan Chandra Roy. The structured structure of this habitus is the character of the doctor as being a virtuous individual with unassailable motives working toward the healing and betterment of society. The structuring structure is the idealized portrait of how a doctor must behave which includes ancient ideals combined with modern examples.

The generative principle of this habitus is compatible with either a fully naïve patient who takes the doctor's advice as is, or a patient with similar cultural capital capable of grasping an accurate even if simplistic image of the doctor's understanding of their disease. It assumes extensive dialog with the patient and appropriate expansion of medical care. However, the parameters of the patient population and the healthcare ecosystem are incompatible with these structuring structures. The appropriate expansion of the healthcare that keeps pace with the growth of the population is missing. The number of doctors present are severely lower than the number required to enable more doctor-patient interaction. The distance in the cultural capital and educational attainment status between the doctors and their patients is wide. The motives of the doctors are constantly questioned by journalists who also perhaps lack the cultural capital required to develop a nuanced understanding of human disease and medical treatments. Finally, there are some bad actors (doctors) whose malpractice may end up adversely affecting the symbolic and social capital of the doctor population as a whole. This habitus (both its structure and its generative principle) are fundamentally incompatible with the reality of the practice of medicine in Government hospitals in West Bengal. As a system of classification this habitus is incompatible with the field of practice of the doctors interviewed as it cannot adequately accommodate the distance in social space between the doctors and the patients. To effectively classify patients as those requiring fiduciary care or those needing to be equal partners, this habitus needs to operate in a space within which either the social capital of doctors is higher (enabling fiduciary care) or a space within which the cultural capital of the patients is higher (enabling equal partnership).

The second habitus structures the doctor as an agent coping with the circumstances within which healthcare is delivered in West Bengal Government Hospitals. Its generative principle is rooted in compromise and pragmatism—how does one cope with the challenges extant in the current healthcare ecosystem? It prioritizes skill development toward efficient handling of patients. Instead of prioritizing the communication of a fully accurate understanding of the disease specific to each patient's educational and cultural attainment level, it focuses on working through larger numbers of patients. Instead of attempting to provide complete comprehensive biopsychosocial care to each patient encountered, it nudges the doctors to hone the skills needed to provide effective resource-limited care. Instead of developing reasoning skills (or gaining communication training as part of the cultural capital) to mollify irate patient relatives, this habitus structures itself to increase referrals (from suburban hospitals) to bigger centers with more facilities, and presumably more security coverage. The structure of this habitus is a set of directives imposed on the doctors by the crushing patient load—each directive implying the setting aside of a particular healthcare objective in order to distribute the healthcare resource to a larger number of patients. This habitus is borne out of the social space of the hospitals and the populations associated with them; therefore, it is compatible with the healthcare ecosystem.

This second habitus performs well as a system of classification within this healthcare system. It allows for picking a course of action as it always reverts to its generative principle—how to process and care for the largest number of patients possible. Due to the low economic capital of the patient population, the doctors also understand that some of these patients need to be seen in a government system because they cannot afford private care. This habitus will always classify patients based on the time necessary to take care of their proximate biomedical concern, rather than their comprehensive biopsychosocial needs. The data on time spent with each patient bears out the prevalence of this habitus.

However, as a habitus whose structure is a set of compromises and whose structuring structure is a series of unfulfilled objectives and the omnipresent act of compromise inherent in medical practice in this milieu, it fails to provide satisfaction to the doctors, nurses, and sometimes patients as well. Some of my data reveal a lack of job satisfaction amongst the doctors and nurses interviewed. Junior doctors interviewed in both metropolitan and suburban sites indicated dissatisfaction with their pay and the associated physical and mental stress involved in the job. Job satisfaction of the doctors is critical for retention given the low doctor-patient ratio. I argue that job satisfaction is an important component of the embodied cultural capital associated with being a doctor, and my data indicates that 50% of the doctors interviewed had family members who were doctors. In order to ensure continuous doctor supply, it is important to ensure the steady supply of embodied cultural capital amongst the doctor population. Deep dissatisfaction with the job owing to a social space consideration driven habitus whose structured structure is a set of least bad choices and whose structuring structure is rooted in making the least bad choice erodes at this embodied cultural capital and potentially affects the supply of new doctors.

Therefore, neither of the extreme habitus are compatible with a sustainable healthcare ecosystem which responds to the constraints present. One can assume that the habitus of the practicing physicians lies somewhere between these two extremes—it is neither fully idealistic nor ruthlessly pragmatic. The incongruity between the habitus of the doctors and social field within which they practice precludes instinctual and smooth interaction and promotes friction. This friction often takes the form of verbal abuse, threats of physical abuse or outright physical violence directed at them. An analysis of the patient habitus within this social space elucidates further sources of friction which perpetuate the deteriorating relationship and corresponding violence.

#### Patient respondents

Patient respondents were identified and recruited *via* snowball sampling after doctor respondents identified them as patients. This was done to ensure that patients are approached rather than their dependents. Sampling patient respondents by approaching patients within the Government hospitals was not performed because Government hospital corridors and outdoor waiting areas are often populated by multiple patient family members and friends (pre-COVID-19). This sampling could have led to non-patients opting to self-identify as patients and may have led to inappropriate data. Thus, the bias introduced by snowball sampling and doctor identification was tolerated to ensure that all the patient respondents were bona fide patients.

A majority of patients 77.42% stated that doctors demonstrated a warm and friendly attitude toward them, 19.35% said that doctors maintained a warm, friendly, and respectful attitude toward them, especially when they were senior citizens. In 3.22% asserted that the doctor was in a hurry. Only 6.06% of patients were very satisfied with nurses' efficiency, 15.15% were somewhat satisfied with their skills, 9.09% expressed neutral views, and 69.70% expressed they were not sure about the nurses' skill and efficiency. The patient population showed some doubts regarding the training, i.e., the cultural capital of the nurses. Some of the patients may also not have had interacted with the nurses because they were seen in the outpatient department, and those typically involve short one-on-one interactions with the attending doctor with minimal nurse involvement.

The analysis of the doctor respondent data and their habitus would presume that patients would indicate high levels of dissatisfaction with the care they received. The patient respondents indicated that the treatment that they received was better than private hospitals (these are higher in the hierarchy of social space than the Government hospitals). Admitted patients especially appreciated doctors' behavior toward them and were satisfied by the medical care that they received. In general, patients asserted that they tried their best in following doctors' instructions. None of the patients indicated that they gave doctors excuses of religious or cultural barriers for not being able to follow particular care instructions. Although, doctors indicated such barriers being posed by patients.

Therefore, from a limited patient point of view we see cause for satisfaction rather than friction in the doctor-patient relationship. Patient dissatisfaction with the healthcare ecosystem is perhaps derived from the cost and availability of medications. Despite over 80% of the patients using the Government Hospital system, West Bengal has some of the highest per capita private medical expenditure amongst states in India. West Bengal had some of the highest out-of-pocket expenses in the country as well (Roy and Gupta, 2011; Bose and Dutta, 2018). This issue arises because of the scarcity of subsidized medications within Government hospital pharmacies driving patients toward unsubsidized private pharmacies which can cost ten times more (ET Online, 2018).

“*This disposition, always marked by its(social) conditions of acquisition and realization, tends to adjust to the objective chances of satisfying need for desire, inclining agents to ‘cut their coats according to their cloth', and so to become the accomplices of the processes that tend to make the probable a reality.”* (Bourdieu, 1990a, p. 65).

Conceptualizing the habitus of the patients helps elucidate some of their thought processes linking their low individual position in the social space and the violence directed at healthcare personnel. Construction of the habitus was performed by integrating patient narratives; secondary source data on out-of-pocket medical expenditures and Government-provided health insurance schemes in West Bengal; differential medication pricing policy; and doctor's responses on schemes to pay for patient care. Patient narratives detailed skipping doses of necessary medication due to unavailability of the prescribed medication in the Government hospital subsidized on-site pharmacy; inability to use Central and State Government insurance schemes—Rashtriya Swasthya Bima Yojana (RSBY) and Swasthya Sathi respectively to pay for dependent's primary care; intimidation and inhibition of patients to go through the bureaucracy required to avail or renew Central and State Government insurance schemes; and having to forego an entire day's salary to come to the hospital. Secondary data on out-of-pocket expenditures included literature analyses of medical expenditures in India; press reporting on RSBY and Swasthya Sathi; and press reporting on differential drug pricing in West Bengal (Roy and Gupta, 2011; Prinja et al., 2012; PTI, 2015; Sheikh et al., 2015; Bose and Dutta, 2018; ET Online, 2018; Dash and Mohanty, 2019; Mishra and Mohanty, 2019). Finally, 70% of the doctor respondents indicated that patients could avail either RSBY or Swasthya Sathi to pay for treatment, but none of the patients had availed these schemes, and these schemes also do not cover outpatient care (Chakraborty, 2020), therefore causing patients to depend on their personal economic means to cover outpatient care associated costs.

The structured structure of the habitus is the understanding of which of the basic necessities of life is expendable in the short term to pay for acquisition of expensive medications or care not obtained at the Government hospital. The structuring structure of the habitus derives from having suffered the consequences of foregoing a basic necessity of either food, clothing, or shelter to pay for personal care or the care of dependents. Their prior experience of suffering informs their understanding of which particular necessity is expendable at the moment to survive. With this habitus, any treatment received which did not involve foregoing a basic necessity of life is likely to have been met with happiness and gratitude. This perhaps explains why patient respondents were content with the care they received and expressed gratitude regarding their communication with doctors, while doctors did not reciprocate this satisfaction with their patient interactions. When inquired about assault against doctors, they implicated third parties (unknown people) lurking around hospitals to generate unrest and turmoil following any unsuccessful medical emergencies. The patient respondents showed no indication of dissatisfaction. However, for the patient population at large, it can be hypothesized that having a habitus built on making choices between medicine and life necessities perhaps leads to dissatisfaction with the healthcare ecosystem and subsequent hostility directed on its most recognizable and proximate agents—doctors and nurses.

#### Nurse respondents

Nurses stand between patients and doctors in the social space in terms of economic, cultural, and individual social capital. Looking at the job satisfaction of the nurses, it appeared that the nurses in the suburban site were likeliest to rate their work atmosphere as stressful (80%) compared to nurse respondents in Kolkata, a minority of whom (32%) rated their work environment as stressful. On average nurses in Kolkata performed more night shifts, but some nurses at the suburban site individually performed between 8–10 night shifts per month (27%). Within the suburban site, 15% of the nurses reported that the doctor's attitude toward them was commanding, while at the Kolkata sites, 20% reported a commanding attitude. Nurses at the suburban sites overwhelmingly reported that they could not spend adequate time with their families (85 vs. 30% in Kolkata). This maybe because at the suburban sites, nurses live in a dormitory-like facility adjacent to the hospital as their hometowns are too distant to effectively commute from.

The most surprising finding came from enquiring into the motivations of the respondents to become nurses. All the women interviewed wanted this profession to become self-reliant. Women in both the suburban and metropolitan areas were driven by the impulse of gaining financial autonomy. Counterintuitively, their families supported this decision made by their daughters, even amongst respondents at the suburban site. The reason these women chose nursing was because nursing provided a guaranteed Government job offer once the minimum eligibility criteria were fulfilled. This is indicative of other factors like the acute shortage of nurses in India (Gill, 2011), and its perception in India as a women's profession still devoid of male contestation (Minocha, 1996; Times News Network, 2016). These factors came together to establish professional nursing as an easily available, stable, and guaranteed pathway to economic independence. The public image of nursing as a noble profession and as a women's profession (Times News Network, 2016) also serves to delineate it. This image eliminates the hesitation associated with women stepping out of the boundaries of the private sphere and into the public domain. This is also evident in the data as most of the nurse respondents' families do not object to their night duties (81% do not object at the suburban site compared to 54% at the metropolitan site). This empowerment narrative in the nurse respondent data provides a convenient starting point to consider the habitus of the nurse and how it can bridge the gap between the two doctor habitus and provide a sustainable non-confrontational path forward for doctor, patient, and nurse relationships.

The generative principle of the nurse habitus is the performance of proscribed duties for economic independence and fulfill their mission as healers. Their role as independent women in the workplace working toward healing people also provides them with embodied cultural capital. The structured structure of their habitus revolves around faithfully executing doctor's instructions for patient care. As they expect to be given instructions by doctors, only a minority consider the doctor's attitude toward them as commanding. Their habitus, while still driven by economic need (similar to patient's habitus) and pulled toward healing in overburdened circumstances (similar to doctor's habitus), is also driven by upliftment and empowerment. Maybe their habitus can help bridge the divide between doctors and patients in the social space hierarchy.

## Discussion

Understanding how the four habitus (two doctor habitus, and nurse and patient habitus) coexist helps make sense of the reasons for the violence and lends context toward wider issues. The doctors are stuck between wanting to provide the best care they can provide and helping the greatest number of people. The nurses are attempting to dutifully provide care as instructed and become empowered citizens. The parsimonious patients are attempting to care for their health but are constantly in fear of the next medical expense that may lead to financial ruin. These habitus are operating in two social spaces, one in which the secondary and tertiary care facilities are situated and one in which their agents (doctors, nurses, and patients) are situated. Into this mix, the politicization of healthcare and health policy introduces an external forcing element which serves to escalate extant tensions.

Consider the hypothetical in which an adverse outcome of a disease has occurred. The doctor, operating using the pragmatic habitus may not have had the time or opportunity to observe the patient's dependents' state of mind. The patient's dependents meanwhile do not fully comprehend that the adverse effect was not a result of the doctor's inability to provide care, but a probable disease outcome. They may also blame the nurses due to the higher position of the nurse in the individual social space for not providing adequate care. The patient habitus may now classify the Government hospital not as a benevolent institution providing subsidized care, but as an institution providing substandard care because of its lower position in the social space. The reclassification now turns the Government hospital from a place of gratitude to a place of ire. The patient families may then want to express their hostility at the hospital. Local politicians who need the patient population social capital, and whose habitus of seeking political prominence are forged through the violence inherent in local electoral politics seize this opportunity and mobilize mobs against the hospital causing physical violence. Using a Bourdieu-ian lens enables us to understand each of the populations and the social space and understand why this dysfunctional healthcare system persists and why when faced with an adverse outcome could quickly morph into a place of physical violence. The COVID-19 pandemic provides another example to understand the sequelae of an adverse outcome given these habitus operating within these social spaces.

The next step is to build on this study to understand how COVID-19 might have changed or aggravated the social dynamics and the social spaces associated with West Bengal Government healthcare. In the backdrop of COVID-19 pandemic, a priori, jingoistic adulation for doctors in combination with fear driven by NIMBYism and stigmatization was observed (Roy, 2020). There is ample praise for the doctors and the work they are doing and their challenges as long as people don't have to interact with frontline healthcare workers in a non-medical setting. The broader population (consisting of patients, dependents, and potential patients) do not want the healthcare workers in their vicinity. For example, when doctors and nurses attempted to rent housing during the pandemic, they were repeatedly denied or even evicted for COVID-19-associated fears (Ellis-Petersen and Rahman, 2020). A deeper analysis of the patient habitus and its method for classifying doctors and nurses as either healthcare heroes or vectors of a deadly virus can help understand this dichotomous perception. This analysis may help us understand why and how patients are attempting to generate this abstract infallible deified idea of a doctor but not wanting to engage with fallible human doctors and nurses.

This analysis has several limitations that may be addressed through future work. While this attempt at theorization was based on a broad empirical study of all three groups, more detailed analysis should be performed on each group in particular. The semi-structured interview format allowed for garnering opinions on a wide range of topics—ranging from social appreciation of healthcare work and interactions to satisfaction with current workplace state. This broad study could be built upon by incorporating either ethnographic or deep qualitative fieldwork focused on smaller sets of issues (administrative dissatisfaction, collegial interactions, deconstruction of a specific instance of violence). The qualitative works may have smaller numbers of respondents but may provide additional promising lines of research to help understand the scenario to eventually provide normative suggestions to improve the relationship and alleviate the violence.

Theorization of the problem starting with other social science perspectives may provide additional insights beyond the scope of this work. The sociological concept of roles and role conflict as laid out by Mead, Merton, Linton, and others (Hindin, 2007) may provide different insights into the problem. The broad sociological concept of roles may be utilized to understand the actions of the doctors as they switch roles between being providers of comprehensive biomedical care to each patient and being healthcare workers attempting to deal with unduly heavy patient loads in a limited time. Nurses switch roles between being aspirational empowered women using their training to enter the Indian middle class and being members of their community who are expected to be able to attend to all their family obligations. Additionally, the difference in the education levels of doctors, nurses and patients implies that the sociolinguistic concept of code-switching can be used to analyze how medical information and diagnoses are communicated differently across different groups. Role theory, dramaturgical sociological analysis, and theories of interactionism maybe used to alternatively analyze some of the data collected to gain different insights into the workplace interactions in West Bengal Government Healthcare. Further studies could extend the fieldwork performed with new fieldwork capturing role switching and role conflicts within each of the populations studied.

Extending this study to focus exclusively on the core violence rather than the interactions could build a theorization based on the perspectives of criminology (Henson, 2010). Henson applies the theories of situational crime prevention to provide normative suggestions to reduce violence in emergency departments. Similar theories maybe used to understand the violence occurring within West Bengal Government hospitals. Fieldwork focusing on the violent actors would extend some of the interaction-based conflict presented in this study. This new fieldwork could be analyzed with criminology theories to understand how the perpetrators of hospital violence are recruited into harming a hospital, including those in which they themselves receive care. In interviews with the press, a senior doctor from Pandua, a suburban area in West Bengal alleges that he was beaten up by a mob armed with metal stands used to hang bottles of saline in hospitals by people in his community including some whose births he had supervised (https://www.youtube.com/watch?v=joH7hAKUWm0, 00:03:30–00:08:00). A criminology-based theorization can help elucidate the sociology of such interactions.

Theorization utilizing the concept of weak ties and strong ties may elucidate the mechanism by which dissatisfaction with healthcare outcomes spreads amongst the patient population (Granovetter, 1973; Small, 2013). Multiple doctor respondents used the term “*negative prochar beshi*” to describe how negative outcomes from treatment are magnified amongst the patient population compared to positive outcomes. Applying the concepts of useful information flow along lines of weak ties, and the tendency to confide in people with whom a weak tie is shared, one can hypothesize that the weak ties that bind patient dependents to local politicians may be responsible for escalation into mob violence. This fieldwork provides information about social ties and community ties present in the population. A further work may build on this concept to clarify the role of strong and weak ties in engendering violence in the West Bengal Government Hospital system.

Theorization utilizing Marxist perspectives may also be used to understand this situation through the lens of class conflict and class solidarity (Gould, 2018). Gould posits that the notions of social movement solidarity have been underutilized in the study of healthcare and structural injustices within healthcare. While this paper alludes to the structural factors affecting the modal suffering of patients, nurses, and doctors, I hypothesize that the concepts of solidarity maybe utilized further to theorize the interactions observed. Traditional analyses of doctor-patient relationships sort the doctors and the patients into separate classes having internal solidarity. I hypothesize that the state of West Bengal government hospital interactions could be understood by sorting doctors and nurses into a traditional “labor” class, while sorting the health ministry and administration into a traditional “manager” class. This Marxist form of classification could shape the nature of future fieldwork and provide additional insights into the structural violence inflicted on the patients and the working conditions of the doctors and nurses.

Therefore, respect, care, violence, and a repressed fear induced by the COVID-19 pandemic appear to exist on a continuum within the West Bengal Government healthcare ecosystem. Bourdieu's expansive social theory augmented with other social science perspectives can help elucidate this continuum.

## Data availability statement

The datasets presented in this article are not readily available because the dataset consists of qualitative interview responses and may contain information that indirectly identifies the respondents. The dataset has been de-identified, but long-form interviewing may reveal sufficient identifiable information. Requests to access the datasets should be directed to TS, tasarkar@ucsd.edu.

## Ethics statement

The studies involving human participants were reviewed and approved by University of Chicago Social and Behavioral Sciences Institutional Review Board. Written informed consent for participation was not required for this study in accordance with the national legislation and the institutional requirements.

## Author contributions

TS conceived the work, performed field interviews, analyzed data, wrote the manuscript, and approved the submitted version.

## Funding

This work was supported by the University Grant Commission and the Ministry of Human Resource Development of India and the University of Chicago Dean of the Social Sciences Research Award.

## Conflict of interest

The author declares that the research was conducted in the absence of any commercial or financial relationships that could be construed as a potential conflict of interest.

## Publisher's note

All claims expressed in this article are solely those of the authors and do not necessarily represent those of their affiliated organizations, or those of the publisher, the editors and the reviewers. Any product that may be evaluated in this article, or claim that may be made by its manufacturer, is not guaranteed or endorsed by the publisher.
