# Supplementary material for: Harnessing Bourdieu's social theory to understand the deteriorating doctor-patient-nurse relationship in West Bengal government hospitals
Source: Front Sociol. 2022 Oct 6;7:938734. doi: 10.3389/fsoc.2022.938734 (PMC9584649; doi:10.3389/fsoc.2022.938734)
Supplement: Supplementary file 1 [file Data_Sheet_1.PDF]

Harnessing Bourdieu's Social Theory to Understand the Deteriorating Doctor-Patient-Nurse Relationship in West Bengal Government Hospitals

Tannistha Sarkar[1]

[1]Department of Sociology, University of California San Diego, 9500 Gilman Dr., La Jolla, CA 92093. United States of America.

*** Correspondence:**
Tannistha Sarkar
tasarkar@ucsd.edu

Keywords: Workplace Violence, Government Hospitals, Healthcare Workers, West Bengal, India, Habitus, Forms of Capital, Social Space.

Supplementary Information – Interview Schedules

APPENDIX

Semi-structured interview schedule for doctors

Socio-Demographic profile of the doctors

1. Name of the Doctor:
2. Age:
3. Sex:
   a) Male b) Female
4. Caste:

   a) General b) SC c) ST d) OBC

5. Nationality:

6. Marital Status:

a) Single b) Married c) Separated d) Divorced

e) Widowed

7. Medical Qualification:

a) M.B.B.S. b) M.D/M. S c) D. M/M.ch.

8. a) How much is your monthly income?

i) Rs. 40,000-50,000

ii) 50,000-60,000

iii) 60,000-70,000

iv) 70,000- 80,000

v) 80,000-90,000

vi) 90,000-1, 00, 000

viii) Above 1, 00, 000

b) How much monthly income ensures an acceptable standard of living for you?

9. Were there doctors among your parents, grandparents, or in your wider family?

i) Several

ii) Yes, there was (one)

iii) No

iv) Doesn't know

10. What is your current position in the Hospital?

i) Intern

ii) House Surgeons

iii) Junior Residents

iv) Senior Residents

v) Consultants

vi) Others

11. Which department of the hospital are you associated to?

12. Experience at your present position:

i) < 1 year

ii) 1-3 years

iii) 3-5 years

iv) > 5 years

Objective 1: To understand the perceptions and expectations of the doctors and patients about the role of the other in the government hospitals in West Bengal.

13. With the rapid progression of medical knowledge, diagnosis and treatment, the practice of medicine has shifted from a paternalistic model toward one of collaboration between doctor and patient. Has this shift been adopted by all patients coming from diverse socio-economic background?

14. Do you think that a doctor's communication style be same for all kinds of patients coming from differing backgrounds?

15. What according to you should be the patients' role in getting cured?

16. Why do you think doctors are more and more getting ensnared in the 'blame culture'?

17. What would you say some of the unrealistic expectations of society are which when not fulfilled, leads to unreasonable dissatisfaction on the part of the patients?

i) Thinking that the Physician will always know the exact diagnosis at first consultation and start treatment immediately.

ii) Ability to call the Physician 24hrs a day for any problem

iii) There is always a vested interest or purposeful negligence on the part of the doctor, in case of failure of treatment.

iv) All the above.

18. The doctor's status has to be more exalted than his patients. What do you think of this statement?

i) Strongly agree

ii) Somewhat agree

iii) Strongly disagree

iv) Somewhat disagree

v) Any other opinions you want to express

Objective 2: To analyse the areas of satisfaction and dissatisfaction related to the interaction and mutual trust between both doctors and patients.

19. a) How many patients do you see per day?

b) Does the hospital regulate the number of patients that you see per day?

c) How much time are you able to assign to each patient?

20. How much freedom you have in making clinical decisions that meet patients' needs?

21. a) How much do patients adhere to your advice?

b) Do you think demographic characteristics of patients like age, gender, educational qualification and socio-economic status determine the rate of patients' compliance?

22. Which reasons would you hold responsible for patients' non-adherence to medical advice?

i) Misunderstanding

ii) Forgetting

iii) Ignoring

iv) Distrust over Physicians

v) Confusion arising out of consulting more than one doctor at the same time for the same ailment.

vi) Complexity of treatment regimens (complex dosing schedules)

vii) Reluctance in modifying existing way of life.

viii) Lack of familial or social support.

23. Non-adherence/compliance on the part of patients can pose risk to the patients' life, and also render the treatment ineffective and incur greater medical costs. In addition to this, does non-adherence/compliance also prove to be frustrating for the doctor? Which of the possible reactions are exhibited by the doctor in face of such non-compliance?

i) Discouraged

ii) Sad

iii) Irritated/Angry

iv) Helpless

v) Other responses

24. Does the emphasis on the participation of patients in the treatment process invoke a sense of loss of respect in the doctor?

Objective 3: To study what determines and affects the communication styles between doctors and patients and compliance patterns of patients:

25. a) Do you think the age of the patient is an important factor to be kept in mind while communicating with patients?

i) Yes ii) No iii) Maybe

b) Do older patients have lower desire for medical decision-making as compared to younger or middle-aged patients?

26. a) Do you think it is a good idea for the doctor to give information related to the illness to adult patients?

i) Yes ii) No iii) Depends on the type of illness iv) Depends on the type of patients

b) Do all patients take an interest in getting informed in detail about their illness or are they content with only how you recommend them regarding medicines and the required therapies to get cured?

27. Is it easier and more satisfying to communicate with patients with higher educational background than patients with lower educational background?

28. Due to the extensive medical resources available on the internet, the patients are now very much informed and take informed medical decisions. What do you think of this group of e-patient?

i) Doctors have more informed discussions with their informed patients.

ii) By acquiring the information about one's illness the patient is exhibiting that he/she is participating in the cure process.

iii) The information on the net lacks reliability and can mislead the patient.

iv) Patients who rely too much on online healthcare information, are likely to consume more healthcare than not, which would probably increase the demand for healthcare.

v)Information available on the internet does not really help the patients in the long run because the final/ accurate diagnosis has to be done by the Physician.

vi) You feel a sense of loss of autonomy or that your medical skills are being questioned when the patient comes armed with information and undermines your medical expertise.

vii) Both (i) and (ii)

viii) (iii), (iv) and (v)

ix) (i), (ii) are true, but you have to keep in mind about (iii), (iv) and (v).

29. a) How does the gender of the patient play a vital role in your communication with him/her?

b) For certain gender-sensitive examinations, do you come across patients who prefer a physician of the same gender?

c) Are your patients always accompanied by family members, in case of gender discordance with the physician?

d) In case of physical examinations, do you conduct it yourself always, even when the patient belongs to the opposite gender?

30. a) Do all your patients speak the same language as you do?

b) Are they able to follow your instructions in case they follow a different language?

31. How do you tackle a patient whose strong cultural beliefs pose as an impediment in following your medical advice?

32. How do beliefs in traditional medicines and customs affect the treatment, progression of illness and the relationship between doctor and patient?

Objective 4: To explore the influence of the infrastructure and procedure on the doctor and patient relationship.

33. Type of work you are engaged in:

i) Full-time ii) Part-time

34. a) Have you worked in other hospitals before the one you are currently employed in?

b) Why did you leave the previous place?

35. How many hours do you have to work in the hospital in a day on an average?

36. How many night duties do you have in a week?

37. How would you define your relationship with the hospital administration?

a) Administration listens to physician concerns.

b) You have a good professional relationship with the administration?

c) During the last year, your professional relationship has improved with the administration.

d) Any other point you want to stress upon.

38. Does the hospital have sufficient equipment for modern diagnosis and treatment?

39. Does the hospital have modern operating room facilities?

40. How satisfied are you with the skill and competency of the nursing staff?

41. i. What kind of health facilities your hospital provides?

a. OPD Services

b. IPD Services

c. OT Services

d. SNCU

e. HDU

f. ICU

g. Dialysis unit

ii. What kind of diagnostic facilities your hospital provides?

c) Radiology
   i. X-Ray
   ii. USG
   iii. CT-Scan
   iv. MRI

d) Pathology
   i. Microbiology
   ii. Biochemistry

42. What kind of insurance/ health policies patients avail in this hospital?

Semi-structured interview schedule for nurses

1. Name

2. Age-

3. Gender- Male/Female

4. Caste- a. General b. SC

c. ST d. OBC

5. Religion-

6. Marital Status- a. Married b. Single

c. Divorced d. Separated e. Widow

7. No. of family members-

8. No. of children, if any-

9. Educational Qualification- a. below Matriculation

b. Matriculation c. HS d. Graduate

e. Postgraduate

10. i. Did you undergo special training for this profession?

a. Yes b. No

10.ii. What is the duration of your training period?

11. What is your highest level of nursing education?
    a. Diploma
    b. bachelor's degree

c. Master's Degree
d. Associate degree
e. Doctorate Degree

12. i. What is your current position in this Hospital?

ii. What is your years of experience in your current position?

13. What is your current employment status?
a. Full Time
b. Part Time

14. What time of day do you work?
a. Day Shift – 8 hours
b. Evening Shift – 8 hours
c. Night Shift - 8 hours
d. Day Shift – 12 hours
e. Night Shift – 12hours

15. i. What are your job plans in the next six months?
a. Stay in Current job
b. Leave Current job

ii. Reasons for leaving:
a. Get another position
b. Maternity Leave/Medical Leave
c. Retirement

16. In the past six months have you worked overtime?
a. Yes b. No c. Sometimes

17. Annual Income- a. Below 2,00, 000 b. 2, 00,000-4, 00, 000

c.4, 00,000-6, 00, 000 d. Above 6, 00, 000

18. Annual Family Income- a. Below 5,00,000

b. 5, 00,000-7, 00,000 c. Above 7, 00,000

19. a. Do your family members support your profession?

a. Yes b. No

b. If no, why?

20. Are you the sole earning member?

a. Yes b. No

21. How is your working atmosphere?

a. Good b. Satisfactory c. Stressful

22. Doctors' attitude towards nurses?

a. Respectful b. Friendly c. Commanding
d. Collaborative (Joint Practice)

23. i. Do you have disagreements or conflicts with doctors?

a. Yes b. No c. Sometimes

ii. On what matters do you generally disagree or have conflicts with the doctors?

iii. How do you resolve those disagreements or conflicts?

a. You ignore the issue and pretend that it will go away.
b. Both parties withdraw from the conflict.
c. All points of view will be carefully considered in arriving at the best solution for the problem.
d. Both parties involved will not settle the dispute until all are satisfied with the decision.
e. Everyone contributes from their expertise and experience to produce a high-quality solution.

24. What kind of relationship do you share with other Nurses?

a. It is easy for you to talk openly with other nurses.
b. You receive incorrect information from other nurses.
c. It is often necessary to go back and check the accuracy of information you have received from other nurses.
d. You have an understanding among yourselves and work accordingly.

25. What kind of a relationship do you share with your senior nurses?
   a. They provide the required advice to junior nurses.
   b. They discourage you from taking initiatives.
   c. They take decisions without taking input from other nurses.
   d. They are co-operative and patient and help you to learn your work.

26. Relationship with patients

a. Professional   b. Friendly   c. Emotional

27. Interaction with patients' relatives

a. Consoling   b. Professional   c. Bitter

28. How do patients behave with you?

29. Ratio of number of patients is to one nurse

a. 1:10   b. 1:15   c.1:20   d. 1:25

30. Night duties you are allotted per month

a. 4-6   b. 6-8   c. 8-10

31. i. Do you think the state health department should take specific steps to make the patient-nurse ratio less?

a. Yes   b. No

ii. Do you think your department needs a greater number of nurses?

32. i. Is the infrastructure of your workplace well maintained?

a. Yes   b. No

ii. If it is not well equipped how do you get affected?

iii. How do you react to such a situation?

a. You adjust b. You appeal c. You protest

33. While working with patients suffering from contagious diseases are you provided with sufficient protective measures?

a. Yes b. No

34.Due to your working conditions are you exposed to health hazards?

a. Yes b. No c. Sometimes

35. How far is your workplace from your residence (km) and what mode of transport do you avail to reach to your workplace?

36. Are nurses represented by a union in the hospital you work?

37. Are you able to give quality time to your family members?

a. Yes b. No

38. a. Are your children getting neglected due to your professional demands?

a. Yes b. No

b. If yes, do they grudge for it?

a. Yes b. No

39. Do you always find time to make yourself available at social/family occasions?

a. Yes b. No c. Sometimes

40. Do your family members and relatives accept your absence, if any, in social occasions?

a. Yes b. No c. Sometimes

41. Do you face any objection from your family members for your night duties?

a. Yes b. No c. Sometimes

42. What kind of relationship do you share with your colleagues?

a. Friendly b. Co-operative c. Professional

43. Have you ever faced sexual harassment during your night duties?

a. yes b. no c. sometimes

44. Why did you choose this profession?

Questionnaires for Patients

Socio-demographic profile of the patients

1. Name of the patient
2. Age
3. Sex:
   a) Male b) Female

4. Caste:

a) General

b) SC

c) ST

d) OBC

5. Nationality

6. Marital Status:

a) Single

b) Married

c) Divorced

d) Separated

e) Widowed

7. Educational Qualification:

a) Below Matriculation

b) Matriculation

c) Higher Secondary

d) Graduate

e) Postgraduate

f) Post Graduate and Above

8. Monthly Income:

a) Below 10,001

b) 10,001 – 20,000

c) 20,001 – 30,000

d) 30, 001 – 40,000

e) 40, 001 – 50,000

f) 50,001 – 60,000

g) 60,001- 70,000

h) 70,001 – 80,000

i) 80,001 – 90,000

j) 90,001 – 1 lakh

k) Above 1 lakh

9. Occupation/Profession:

10. Where do you live/ come from?

Objective 1: To understand the perceptions and expectations of the doctors and patients about the role of the other in the Government Hospitals in West Bengal

11. For what kind of ailment have you come to visit the doctor?

12. a) What kind of symptoms were you experiencing?

b) How did you describe them to your doctor?

c) Were you able to explain to the doctor what problems you were experiencing related to your health and in case you didn't know how to describe or were faced with lack of proper words to describe the malady, was the doctor able to come to your rescue by giving you the exact words that will describe your ailment?

d) Did you feel that the doctor understood you and the disorder that you might be suffering from?

e) Was he able to make you confident and hopeful that you will get better?

13. a) Were you accompanied by a family member?

b) Did the doctor consider the presence of the accompanying person and greet that person along with you?

14. a) Are you visiting this doctor for the first time?

i) Yes ii) No

b) If No, then how many times have you visited this doctor before?

15. Who referred you to this doctor?

16. Why did you choose this doctor?

17. a) How long did you talk to the doctor?

b) If this isn't your first visit to this doctor then, what is the length of consultation time you generally have with the doctor?

18. How long did you have to wait for your turn for the consultation?

Objective 2: To analyse the areas of satisfaction and dissatisfaction related to the interaction and mutual trust between both the doctors and patients.

19. a) How was the doctor at making you feel at ease (Being friendly or warm towards you, treating you with respect, and not abrupt or cold)?
    b) Did the doctor listen to you carefully, not acting bored or ignoring what you have to say?
    c) Did he interrupt you while you were talking and asked thoughtful questions only when you were finished speaking?
    d)Did he encourage you to ask questions and answered your questions patiently?

20. When you go for medical care are the doctors careful to check everything and do, they take care to cause as less discomfort or pain as possible while examining you?
21. a) Does the doctor tell you before a physical examination what she/he is going to do?
    b) Does your doctor always talk about what he/she formed out after examining you?

22. a) Was the doctor able to explain your illness to you in a way that you will understand?

b) Did the doctor use too many medical terms that you did not understand or could not retain after your medical consultation?

23. a) Did the doctor write you a prescription and took the time to explain to you the dosage of your medication?

b) Was the doctor's handwriting in the prescription legible?

Objective 3: To study what determines and affects the communication styles between doctors and patients and compliance patterns of patients.

24. Does the Doctor get angry at you easily when you do not understand his instructions?

25. a) When you cannot comply with a particular type of care process because of some because of some cultural barriers does the doctor take that into consideration?

b) Does he suggest alternative care treatments?

26. a) Does your doctor give you a lot of information even when you have not asked for it?

b) Does that make it difficult for you to remember everything or you appreciate the doctor for sharing all the information?

27. a) The medications that the doctor advised you to take, were they cost-friendly/ affordable?

b) Do you think that you are getting the medical care that you need without being financially set back?

28. a) Did the doctor ask you to get medical tests to diagnose your illness?

b) How many medical tests did he prescribe at once?

29. a) Do you follow the treatment procedures as directed by your doctor?

b) Have you been taking all the medications as has been prescribed by your doctor?

i) Yes ii) No

c) If No, what would you say are the reasons behind not taking medicines?

i) Simply forgot

ii) Had too many pills to take

iii) Were busy with other things

iv) Felt like the drug had side-effects

v) Ran out of pills, and forgot to buy them

Objective 4: To explore the influence of the infrastructure and procedure on the doctor and patient relationship.

30. Are you visiting this hospital for the first time?

31. Why did you choose this hospital?

i) It was in your locality

ii) A specialist was available

iii) My Physician practices here

iv) More familiar with this hospital

v) Cost-friendly

vi) Other reasons

32. Do all your family members come to this hospital as well for medical treatment?

33. How satisfied are you with the skill and competency of the staff?

i) Very satisfied

ii) Somewhat satisfied

iii) Neutral

iv) Somewhat dissatisfied

v) Very dissatisfied

vi) Not sure

34. Does the hospital have equipment for modern diagnosis?

i) Yes

ii) No

iii) Not sure

35. a) Are you able to get your medical tests done in this hospital?

i) Yes ii) No

b) If yes, then does the hospital offer speedy completion of medical tests/ examination reports?

36. Does the hospital have modern operating room facilities?

i) Yes

ii) No

iii) Not sure

37. How satisfied are you with the cleanliness of the hospital

i) Very Satisfied

ii) Somewhat Satisfied

iii) Neutral

iv) Somewhat dissatisfied

v) Very dissatisfied

vi) Not sure

38. How satisfied are you with the efficiency of the nursing care

i) Very satisfied

ii) Somewhat satisfied

iii) Neutral

iv) Somewhat dissatisfied

v) Very dissatisfied

vi) Not sure

39. How satisfied are you with the friendliness and courtesy of the staff

i) Very satisfied

ii) Somewhat satisfied

iii) Neutral

iv) Somewhat dissatisfied

v) Very dissatisfied

vi) Not sure

40. How satisfied are you with the cost of healthcare

i) Very satisfied

ii) Somewhat satisfied

iii) Neutral

iv) Somewhat dissatisfied

v) Very dissatisfied

vi) Not sure
